# Impact of rumen microbiome on cattle carcass traits

**DOI:** 10.1038/s41598-024-56603-3

**Published:** 2024-03-13

**Authors:** Yoshiaki Sato, Ruki Sato, Emiko Fukui, Fumiaki Yoshizawa

**Affiliations:** Department of Agrobiology and Bioresources, School of Agriculture, Utsunomiya University, Tochigi, Japan

**Keywords:** Archaea, Bacteria, Microbial communities

## Abstract

Rumen microbes are crucial in the anaerobic fermentation of plant polysaccharides to produce volatile fatty acids. However, limited information exists about the specific microbial species and strains in the rumen that affect carcass traits, and it is unclear whether there is a relationship between rumen metabolic functions and these traits. This study investigated the relationship between the rumen microbiome and carcass traits in beef cattle using 16S rRNA amplicon and shotgun sequencing. Metagenomic sequencing was used to compare the rumen microbiome between high-carcass weight (HW) and low-carcass weight (LW) cattle, and high-marbling (HM) and low-marbling (LM) cattle. Prokaryotic communities in the rumen of HW vs. LW and HM vs. LM were separated using 16S rRNA amplicon sequencing. Notably, shotgun metagenomic sequencing revealed that HW cattle had more methane-producing bacteria and ciliate protozoa, suggesting higher methane emissions. Additionally, variations were observed in the abundances of certain glycoside hydrolases and polysaccharide lyases involved in the ruminal degradation of plant polysaccharides between HW and LW. From our metagenome dataset, 807 non-redundant metagenome-assembled genomes (MAGs) of medium to high quality were obtained. Among these, 309 and 113 MAGs were associated with carcass weight and marbling, respectively.

## Introduction

The demand for animal proteins has increased as the global population continues to grow. Ruminant products, particularly milk and meat, play pivotal roles in global food security. Ruminants possess a four-compartment stomach, with the rumen as the first compartment of the digestive tract. Complex microbial ecosystems in the rumen are composed of bacteria, protozoa, fungi, archaea, and viruses^1^ and enable the anaerobic fermentation of plant polysaccharides, thereby producing microbial metabolites such as volatile fatty acids (VFAs), microbial proteins, and methane^2^. VFAs are absorbed through the rumen epithelium and serve as the primary energy source for the host, constituting 80% of its energy needs^3^. Microbial proteins are assimilated by hosts in the gastrointestinal tract following the rumen and provide more than half of their amino acids^4^. Thus, rumen microbes are essential for transforming human-indigestible plant biomass into human-digestible foods, such as milk and meat. A comprehensive understanding of microbial composition and function is essential for improving the productivity of ruminants.

In addition to carcass weight, beef marbling also has a significant influence on carcass price^5,6^. Therefore, beef marbling is an important indicator for producers owing to its increased economic benefits, and enhancing these carcass traits is essential for beef production. Rumen-related functions, such as microbial degradation and protein synthesis, are associated with these traits, as well as other factors, including genetics^7^, feeds^7^, and blood metabolites^8^. For example, VFAs produced by rumen microorganisms are associated with intramuscular fat (IMF) content in sheep^9^. Additionally, Japanese black (JB) cattle produce high marbling compared to other breeds^10,11^, with taxonomical and functional differences in the rumen microbiome of crossbreeds^12^. Consequently, it has been speculated that the rumen microbiome may be associated with the carcass traits of hosts. Indeed, rumen microbial taxa are associated with carcass weight and beef marbling. Krause et al*.*^13^ reported that marbling scores are positively correlated with the alpha diversity of the rumen microbiome and the relative abundance of the S24-7 family, whereas Actinobacteria are associated with yield grade. Kim et al*.*^14^ demonstrated that some bacterial taxa, such as Verrucomicrobia and *Olsenella*, are associated with marbling in Korean beef cattle. However, these studies did not investigate the microbial species and strains in the rumen that influence carcass traits. Although previous studies predicted functional characteristics using 16S rRNA gene sequencing, there is a paucity of information on whether metabolic functions in the rumen are related to carcass traits^13,14^. Therefore, it is necessary to understand the functional potential of microbial communities in the rumen in relation to carcass traits using metagenomic sequencing.

Here, we investigated the relationship between rumen microbiome and carcass traits of beef cattle and taxonomically and functionally characterized several taxa involved in beef production. Our results identified that methane-producing bacteria and ciliate protozoa involved in high-carcass weight cattle, suggesting that the difference of methane metabolisms in the rumen caused the difference of beef production. Additionally, we found many metagenome-assembled genomes (MAGs) which associated with carcass weight and marbling. This study contributes to the better understanding of the rumen microbiome and the development of the beef production.

## Materials and methods

### Ruminal fluid sampling, DNA extraction, and sample information

Ruminal fluid was collected from 55 JB cattle on a viscera table in a few minutes after evisceration at a commercial slaughterhouse. The rumen samples were filtered through four layers of cheesecloth, transported on dry ice to the laboratory, and stored at -80 °C until use. The samples were thawed and centrifuged at 12,000×*g* at 4 °C for 15 min. The supernatant was discarded and the residues were used for DNA extraction. DNA was extracted from rumen samples following the method described by Frias-Lopez et al.^15^, with a minor modification as reported by Sato et al*.*^12^. The extracted DNA was stored at − 20 °C until required for sequencing. Ethical approval was not required for the current study as animals were killed for commercial purposes unrelated to this research.

The carcass weight and beef marbling data were recorded. The 12-point beef marbling standard (BMS)^16^, which is commonly employed in Japan, was used as an index for evaluating beef marbling. JB cattle were allocated to the following two groups based on carcass weight: high carcass weight (HW; n = 12) and low carcass weight (LW; n = 12). Similarly, the high-marbling (HM) (n = 13) and low-marbling (LM) (n = 12) groups were designated based on cattle BMS scores.

### The 16S rRNA gene sequencing

For all samples (n = 55), the V3–V4 hypervariable region of the 16S rRNA gene was amplified using the Pro341F (5′-CCTACGGGNBGCASCAG-3′) and Pro805R (5′-GACTACNVGGGTATCTAATCC-3′) primers^17^ with Illumina overhang adapters. Amplification was performed under the following conditions: an initial denaturation step at 95 °C for 3 min, followed by 25 cycles of denaturation at 95 °C for 30 s, annealing at 55 °C for 30 s, extension at 72 °C for 30 s, and a final elongation step at 72 °C for 5 min. Subsequently, the samples were indexed using a Nextera XT index kit (Illumina, San Diego, CA, USA) and sequencing was performed using an Illumina MiSeq platform (2 × 300-base pairs).

After sequencing, quality filtering, denoising, and pair-end merging were performed using the DADA2 plugin^18^ in QIIME2 v. 2023.2^19^. Subsequently, a feature table of amplicon sequence variants (ASVs) was constructed. For taxonomic analysis, ASVs were aligned with the SILVA 138 reference database^20^. ASVs taxonomically assigned to mitochondria, chloroplasts, eukaryotes, and those with unassigned kingdoms were removed from the feature table of the 16S rRNA gene sequences. The samples were rarified to the lowest sample depth of 53,476 sequences per sample for diversity analysis. Alpha diversity, including observed ASVs and Shannon diversity^21^, were estimated using the ‘Phyloseq’ package of R^22^. Beta diversity was calculated with a Bray–Curtis dissimilarity matrix at the ASV level using the ‘vegan’ package in R^23^. Non-metric multidimensional scaling (NMDS) plots based on Bray–Curtis dissimilarity were visualized using ‘ggplots2’ in R^24^.

### Metagenome sequencing

Thirty-seven samples that were allocated based on carcass traits were subjected to shotgun metagenomic sequencing. Metagenomic libraries were prepared using the Illumina DNA Prep and Tagmentation Kit (Illumina). All libraries were sequenced on an Illumina Hiseq X platform (2 × 150-base pairs), generating 67.1 ± 18.26 million reads per sample (mean ± standard deviation, SD).

Low-quality reads and adapters were removed using Trimmomatic v0.39^25^. To filter out host contamination, the trimmed reads were aligned to the bovine reference genome ARS-UCD1.2/bosTau9 (GCF_002263795) using BWA-MEM^26^ and the unmapped reads were retained. Subsequently, the filtered paired and unpaired reads were used for assembly with SPAdes v3.15.3, employing the “–meta” option^27,28^. Contigs longer than 1,000 bp were retained for further analysis.

Protein prediction on the contigs was performed using Prodigal v2.6.3^29^ using the “-p meta” option. The predicted proteins from all contigs were combined and clustered using CD-HIT v4.8.1^30^ with a 95% identity and 90% coverage threshold. This resulted in 16,441,181 predicted proteins from rumen metagenomes, which were further clustered into 5,712,223 gene clusters based on the specified threshold. The filtered paired-end reads were mapped to a non-redundant protein database using BamM (https://github.com/Ecogenomics/BamM). Read counts and transcripts per million (TPM) values for each protein were determined using CoverM v.0.4.0 (https://github.com/wwood/CoverM) with the following parameters: ‘–min-covered-fraction 0 –min-read-percent-identity 95 –min-read-aligned-percent 90’. The relative abundance of proteins was calculated based on the TPM values. Clustered proteins were mapped against the non-redundant NCBI protein database using DIAMOND v.2.1.8^31^. For taxonomic classification, the output was analyzed using MEGAN v.6.24.23, with the lowest common ancestor algorithm^32^. For functional analysis, proteins were aligned with the Kyoto Encyclopedia of Genes and Genomes (KEGG) database using KofamScan v.1.3.0^33^, and KEGG Orthologs (KOs) were used for KEGG pathway analysis using KEGG Mapper^34^. Clusters of Orthologous Group (COG) categories of the proteins were predicted by annotating them to the eggNOG v.5.0 database^35^ using eggNOG-mapper v. 2.0.1^36^. Additionally, Carbohydrate-Active enZyme (CAZyme) annotation was conducted using HMMER v.3.3^37^ against the dbCAN HMM v11 database with an E-value threshold < 1e−15 and a coverage > 0.35^38^.

Filtered reads were aligned to contigs using BWA-MEM^26^ for metagenomic binning. Metagenomic binning was performed using MaxBin2 v2.15^39^, MetaBAT2 v2.27^40^, and CONCOCT v1.10^41^. Subsequently, MAGs were optimized using DAS Tool v1.1.4^42^.

The quality of MAGs was assessed using CheckM v1.2.0^43^, followed by collection of “medium-quality” (completeness ≥ 50% and contamination ≤ 10%) and “near complete” MAGs (completeness ≥ 90% and contamination ≤ 5%). Additionally, the “near complete” MAGs that had the 5S, 16S, and 23S rRNA genes, and at least 18 tRNAs were classified as “high-quality” MAGs according to the Minimum Information about Metagenome-Assembled Genome standards^44^. The rRNA and tRNA genes in the MAGs were predicted by Barrnap v.0.9 (https://github.com/tseemann/barrnap) and tRNAscan-SE 2.0 v.2.0.7^45^, respectively. The MAGs that exceeded “medium-quality” were used for further analysis.

These MAGs were dereplicated at a 99% average nucleotide identity (ANI) threshold using dRep v3.2.2^46^. Furthermore, non-redundant MAGs were combined with ruminal microbial genomes (referred to as rumen microbial genomes, RMGs) from previous studies^47–50^ and dereplicated at a 95% ANI threshold to generate representative species-level MAGs using dRep v3.2.2^46^. Read mapping to the MAGs was carried out using BamM (https://github.com/Ecogenomics/BamM), and read counts and TPM values were calculated using CoverM v.0.4.0 (https://github.com/wwood/CoverM). The taxonomical classification of non-redundant MAGs was conducted using GTDB-tk v2.1.1^51^, with GTDB release 207. A phylogenetic tree of MAGs was constructed using PhyloPhlAn v3.0.60^52^ and visualized using iTOL v6.1.2^53^.

Protein prediction in MAGs was performed using Prodigal v.2.6.3^29^. KEGG and CAZyme annotations were performed as previously described. Specific marker genes were considered as indicators for the assessment of acetate, propionate, and butyrate production potential. Acetate production was evaluated using acetate kinase (K00925), phosphate acetyltransferase (K00625 and K13788), and a putative phosphotransacetylase (K15024). Propionate production via the succinate pathway was assessed using genes annotated as succinyl-CoA synthetase (K01902 and K01903), methylmalonyl-CoA (K01847), methylmalonyl-CoA decarboxylase (K05606), methylmalonyl-CoA decarboxylase (K11264), and propionate CoA transferase (K01026). Butyrate production potential was inferred from phosphate butyryltransferase (K00634), butyrate kinase (K00929), butyryl-CoA dehydrogenase (K00248), and acetate-CoA/acetoacetate-CoA-transferase (K01034 and K01035). Polysaccharide utilization loci (PUL) were predicted using PULpy^54^.

### Statistical analyses

Statistical analyses of carcass weight and BMS scores were conducted using one-way analysis of variance. To investigate the differences in diversity between HW and LW, and HM and LM, we performed the Mann–Whitney U test for alpha diversity and permutational multivariate analysis of variance (PERMANOVA) with 9,999 permutations for beta diversity. Differences were considered statistically significant at P < 0.05.

Differential abundance analysis between each group for microbial composition, MAGs, COGs, KOs, and CAZymes was performed using the Wald test within DESeq2 based on the read count matrix^55^. For analysis, p-values were adjusted using the Benjamini–Hochberg method. A adjusted P < 0.05 and an absolute log2 fold change (log2FC) > 1 were considered indicative of a significant difference.

## Results and discussion

### Animal measurements

A summary of the carcass traits is provided in Supplementary data S1. The carcass weight for HW and LW were 617.0 ± 39.59 kg and 488.4 ± 20.46 kg (mean ± SD), respectively, and these values were significantly different (P < 0.01) (Supplementary Fig. S1A). Similarly, the BMS scores for the HM and LM were ≥ 11 and ≤ 6, respectively, and a significant difference was observed between the groups (P < 0.01) (Supplementary Fig. S1B). No significant correlation was observed between carcass weight and BMS (P > 0.05) (Supplementary Fig. S1C).

### The 16S rRNA amplicon sequence

**Figure 1:**
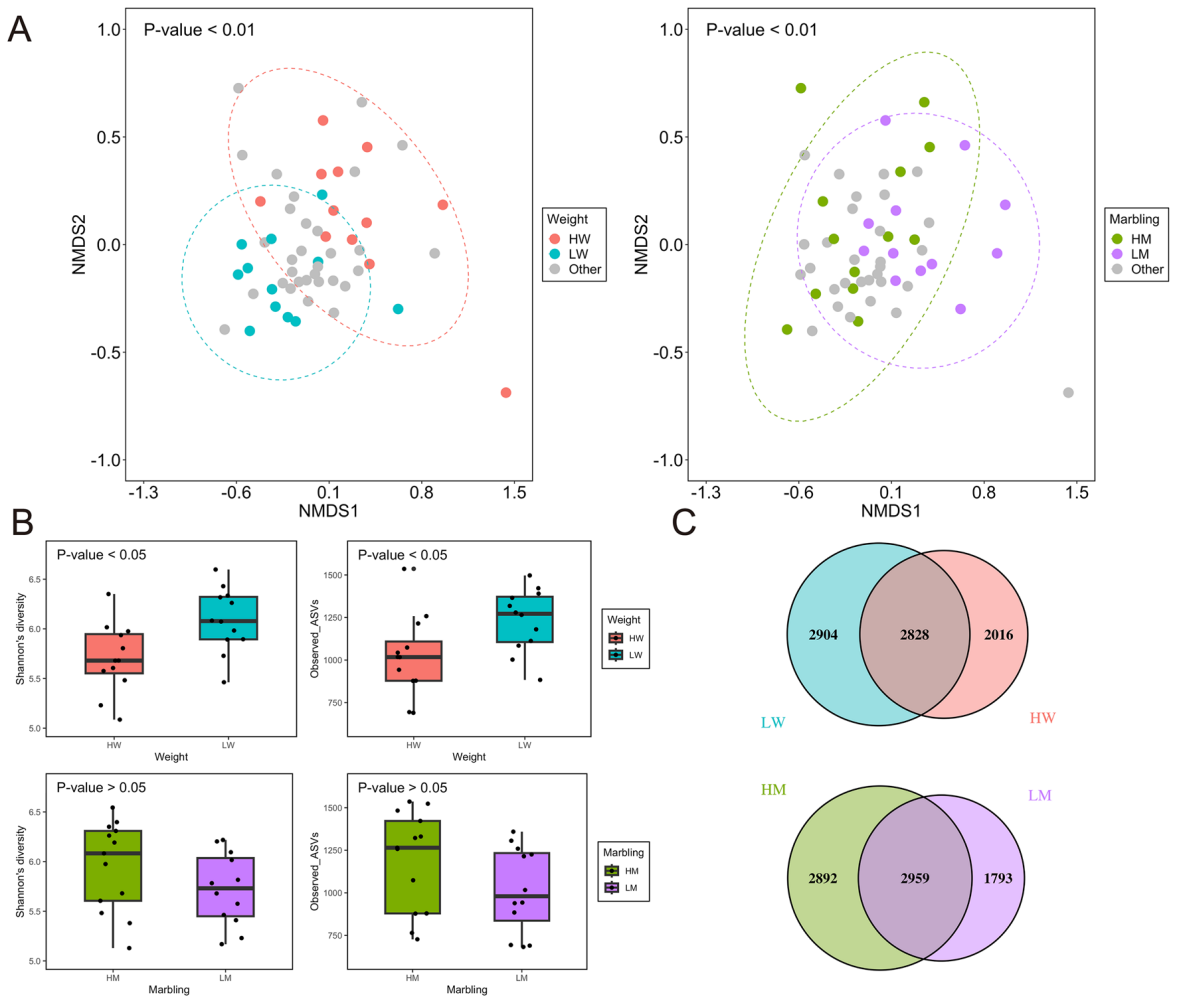
Rumen microbial diversity of animals based on 16S rRNA amplicon sequencing. (**A**) NMDS plots depicting Bray–Curtis dissimilarities based on ASVs. Significance was analyzed using PERMANOVA with 9,999 permutations (P < 0.05). Ellipses represent 95% confidence intervals. “Other” represents samples other than HW, LW, HM, and LM. (**B**) Shannon index and observed ASVs of the rumen microbiota. Significant differences were confirmed using the Mann–Whitney U test (P < 0.05). (**C**) Venn diagram of ASVs among different groups.

The prokaryotic communities between HW and LW (P < 0.01) and between HM and LM (P < 0.01) were separated based on Bray–Curtis dissimilarity at the ASV level (Fig. 1A). Furthermore, we observed a distinct separation in the rumen microbiome between HW and LW at the genus level (P < 0.01), although there were no distinct differences in the microbial diversity between HM and LM (Supplementary Fig. S2). The observed ASVs and Shannon diversity indices of LW were higher than those of HW (P < 0.05) (Fig. 1B). However, there were no significant differences in alpha diversity between HM and LM (Fig. 1B), which is inconsistent with a previous study that investigated the relationship between the rumen microbiome and marbling in Angus steers^13^. Another study reported no significant differences in alpha diversity between high- and low-marbling groups of Korean native beef cattle^14^. These differences may be attributed to the fact that the rumen microbiome varies across breeds^12,56,57^. In total, 2,828 ASVs were shared between LW and HW, and 2,904 and 2016 ASVs were unique to LW and HW, respectively (Fig. 1C). Similarly, 2,959 ASVs were found in HM and LM, with 1,793 and 2,892 ASVs present in LM and HM, respectively (Fig. 1C). Collectively, these results indicated that there are relationships between rumen microbiomes and carcass traits, particularly carcass weight, rather than marbling.

Firmicutes (47.7%) and Bacteroidota (previously Bacteroidetes) (42.6%) were predominant in the rumen samples at the phylum level, which is consistent with previous studies^12,58,59^ (Supplementary Fig. S3A). Additionally, *Prevotella* (17.4%) and *Succiniclasticum* (8.7%) were abundant at the genus level (Supplementary Fig. S3B). These genera are the dominant taxa in the rumen of Japanese Black cattle^12^.

### Protein-based analysis of metagenomic sequencing

#### Taxonomic assignment

Taxonomic annotations were available for 1,936,506 non-redundant proteins (33.9%) at the domain level. The taxonomic composition displayed significant variability among animals, with Bacteroidetes (66.9%) being the predominant phylum, followed by Firmicutes (24.7%), (Supplementary Fig. S4A). Compared to the results of 16S rRNA sequencing, the relative abundance of Bacteroidetes was higher, while that of Firmicutes was lower. In general, shotgun metagenome sequencing can identify significantly more bacterial species than 16S rRNA sequencing^60^. The differences in the resolution between the two methods might lead to different results.

**Figure 2:**
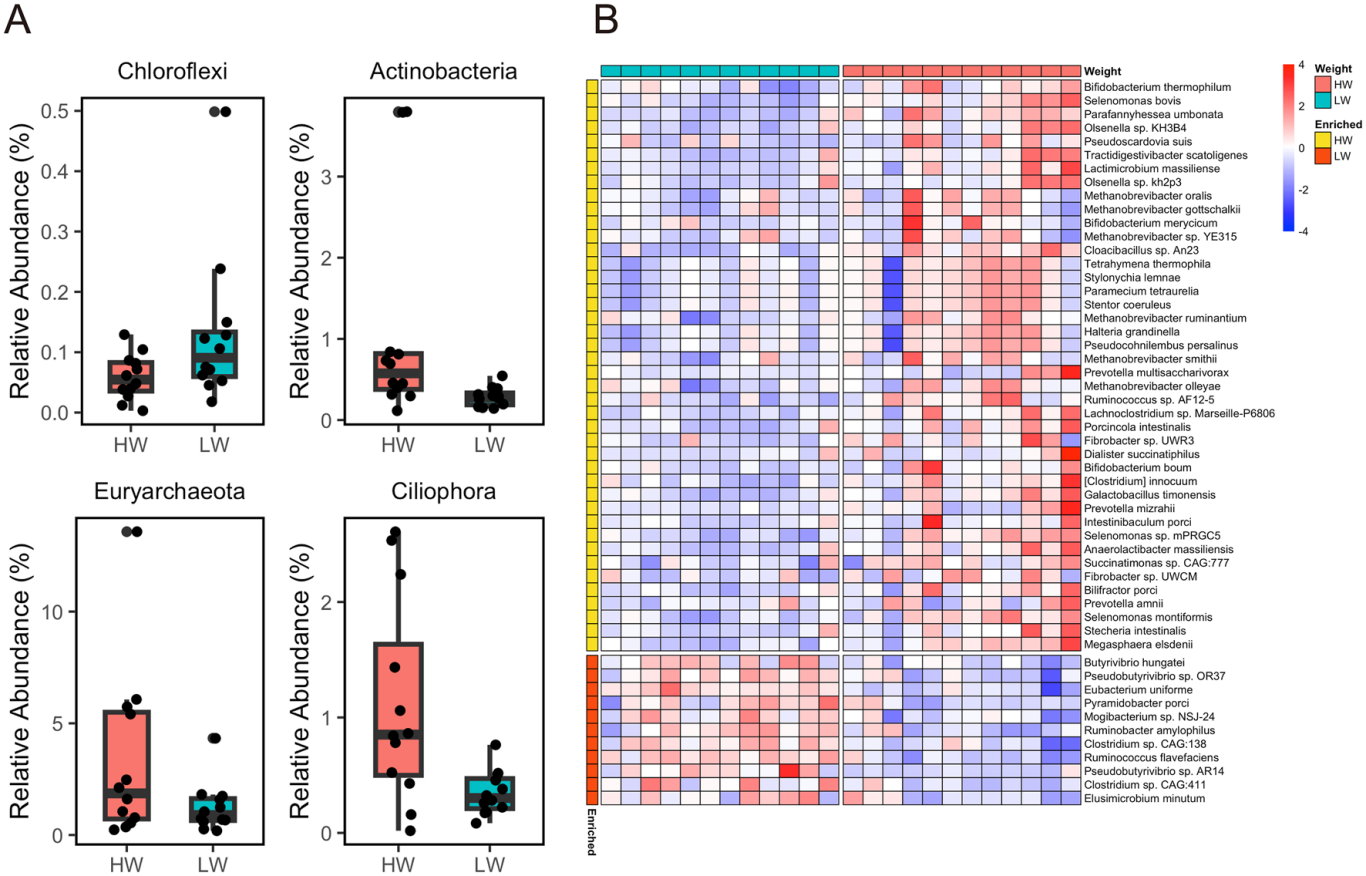
Taxonomic differences between HW and LW groups by protein-based analysis using metagenome sequencing. (**A**) Relative abundances of different microbial taxa at the phylum level. (**B**) The heatmap shows the differential abundance of microbial species based on logarithmically transformed TPM values (log_10_(TPM + 1)). The color code represents the Z-score of the row. Significant differences were identified using DESeq2 (adjusted P < 0.05, |log2 fold-change |> 1).

Comparing HW and LW, Actinobacteria, Euryarchaeota, and Ciliophora were more abundant in HW, whereas Chloroflexi was more abundant in LW (adjusted P < 0.05 and |log2FC|> 1) (Fig. 2A). Actinobacteria are positively correlated with the yield grade of beef cattle^13^. At the species level, 53 species (42 in HW and 11 in LW) showed significant differences between HW and LW (adjusted P < 0.05, |log2FC|> 1) (Fig. 2B). Several ruminal fiber-degrading bacteria, including *Fibrobacter* spp., *Ruminococcus* sp., and *Prevotella* spp.^61^, were enriched in the HW group. In addition, bacteria related to short-chain fatty acid (SCFA) production, such as *Succinatimonas* sp., *Selenomonas* spp., *Megasphaera* sp., and *Bifidobacterium* spp., were more abundant in the HW group. For example, *Succinatimonas* primarily produces succinate^61^ and some *Selenomonas* can utilize soluble sugars to produce lactate, formate, acetate, and propionate^62^. *Megasphaera elsdenii* utilizes lactate to produce butyrate^63^ and is a characteristic bacterial species associated with efficient cattle^64^. Notably, six *Methanobrevibacter* spp., the predominant methanogens in the rumen^65,66^, were more abundant in HW than in LW. Additionally, six ciliate protozoan species, including *Tetrahymena* sp., *Stylonychia* sp., *Paramecium* sp., *Stentor* sp., *Halteria* sp., and *Pseudocohnilembus* sp., were more abundant in HW than in LW. A recent comprehensive analysis of rumen microbiota revealed that ciliate protozoa, except for *Halteria* sp., are associated with methane production^67^. The higher abundance of methanogens in HW cattle strongly suggest that these animals emit higher amounts of methane. Methanogenesis by methanogens is considered the main H_2_ sink in the rumen^68^. The accumulation of H_2_ from bacterial fermentation inhibits microbial fermentation by increasing the partial pressure of H_2_^69^. Cattle with higher carcass weights, through increased methane emissions, may maintain lower H_2_ concentrations in the rumen, creating a more favorable environment for efficient fermentation. Conversely, seven out of 11 species enriched in LW cattle belonged to the class Clostridia, including *Butyrivibrio* sp. and *Pseudobutyrivibrio* spp., which are butyric acid bacteria in the rumen^62^. *Ruminobacter amylophilus*, known for its amylolytic capacity^70^, was more abundant in the LW group. Thus, the abundance of rumen bacteria related to carbohydrate metabolism and SCFA production differed between high- and low-carcass weight animals. No differences were observed between HM and LM at the phylum level and only two species (*Olsenella* sp. KH3B4, and *Tractidigestivibacter scatoligenes*) were more abundant in LM than in HM.

#### Functional analysis

**Figure 3:**
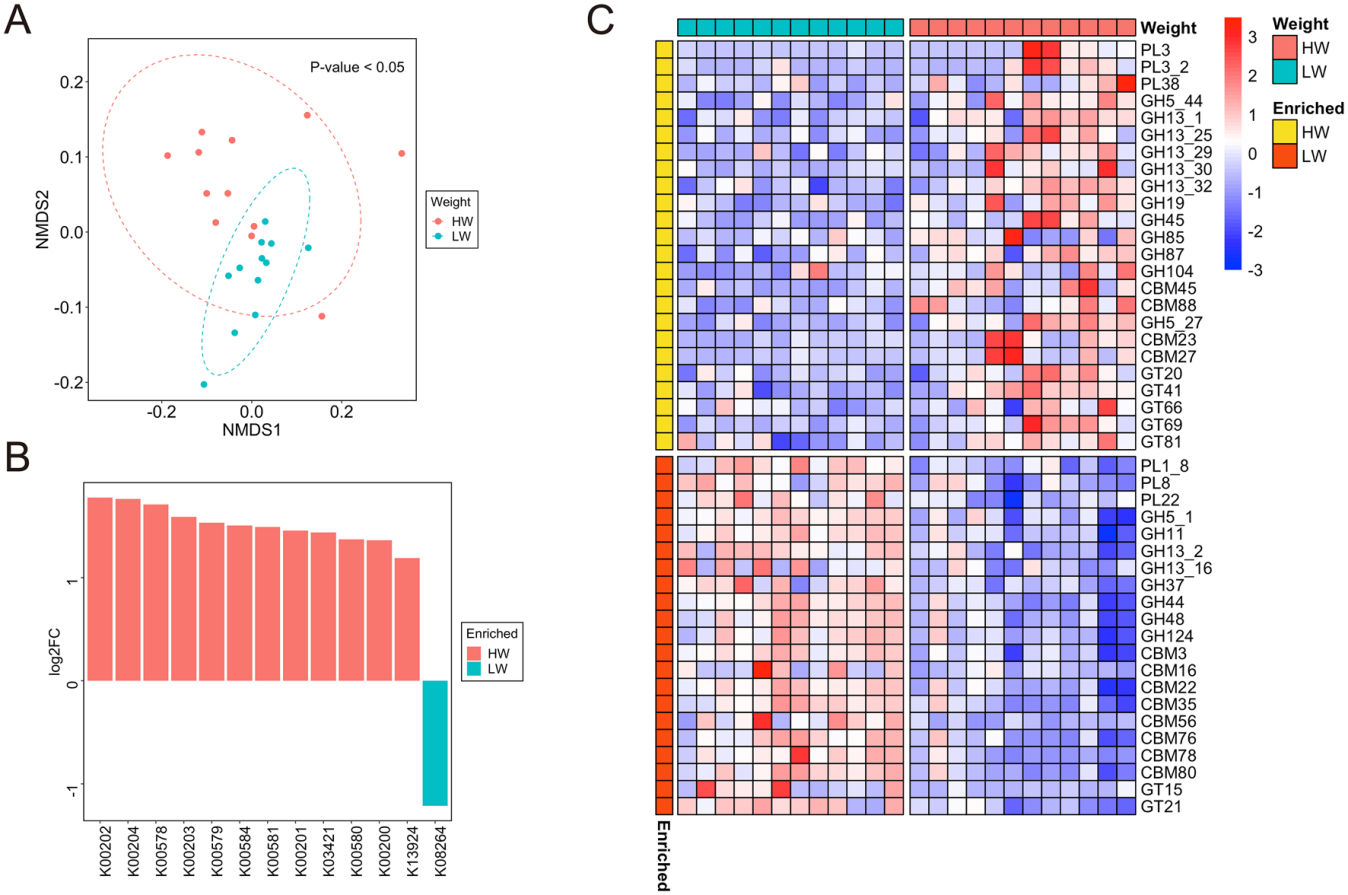
Functional differences in the rumen microbiome between HW and LW cattle revealed by protein-based analysis using metagenomic sequencing. (**A**) NMDS plots depicting Bray–Curtis dissimilarities of KO. Significance was analyzed using PERMANOVA with 9,999 permutations. Ellipses represent 95% confidence intervals. (**B**) Log2 fold change of KOs which related to hydrogenotrophic methanogenesis and show significant differences between HW and LW. (**C**) The heatmap shows the differential abundance of and CAZyme domains based on logarithmically transformed TPM values (log_10_(TPM + 1)). The color code represents the row Z-score. Significant differences were identified using DESeq2 (adjusted P < 0.05 and |log2 fold change|> 1).

A total of 3,766,896 (65.9%), 1,360,793 (23.8%), and 170,308 (2.98%) proteins were assigned to COG categories, KOs, and CAZyme domains, respectively. In contrast to the microbial composition, functional profiles remained stable, as previously reported^71^ (Supplementary Fig. S4B). When comparing the functional characteristics between HM and LM, the NMDS plot based on KOs showed no significant differences among the groups (P > 0.05, PERMANOVA; Supplementary Fig. S5A). Only 12 KOs and four CAZyme domains exhibited differential abundances. Specifically, carbohydrate-binding module (CBM) 37 and glycosyl transferase (GT) 70 were enriched in the HM, whereas glycoside hydrolase (GH) 5_5 and CBM36 were enriched in the LM (Supplementary Fig. S5B,C). This suggested that the microbial functions in the rumen have little influence on marbling. In contrast, NMDS analysis based on the KOs revealed distinct clustering between HW and LW (P < 0.05, PERMANOVA; Fig. 3A). A total of 911 KOs (799 in HW and 112 in LW) showed significant differences among groups (adjusted P < 0.05, |log2FC|> 1), indicating that carcass weight had a strong influence on rumen function and microbial composition (Supplementary Fig. S6A). Notably, 35 KOs related to methane metabolism (ko00680) were enriched in HW cattle (Supplementary Fig. S6B). For example, genes encoding formylmethanofuran dehydrogenase (K00200-204), 5,10-methenyltetrahydromethanopterin hydrogenase (K13942), tetrahydromethanopterin S-methyltransferase (K00578-581 and K00584), and methyl-coenzyme M reductase (K03421), which are involved in hydrogenotrophic methanogenesis, were enriched in HW (Fig. 3B). This can be attributed to the fact that the abundance of methanogens was higher in HW than in LW cattle. Additionally, 14 KOs associated with starch and sucrose metabolism (ko00500), including maltose-6'-phosphate glucosidase (K01232) and trehalose-6-phosphate hydrolase (K01226), were more abundant in HW compared to LW cattle (adjusted P < 0.05 and |log2FC|> 1) (Supplementary data S2).

Similarly, GHs and polysaccharide lyases (PLs) involved in the degradation of plant polysaccharides, such as GH13_1, GH13_32 (α-amylase), GH13_29 (trehalose-6-phosphate hydrolase), GH13_25 (α-glucosidase), GH5_44 (β-galactosidase), GH45 (cellulase), PL3, and the subfamily PL3_2 (pectate lyase) were enriched in HW cattle (adjusted P < 0.05 and |log2FC|> 1) (Fig. 3C). Alternatively, PL1_8 (pectate lyase), PL22 (oligogalacturonate lyase), GH5_1, GH44, GH48, GH124 (cellulase), GH11 (endo-β-1,4-xylanase), and GH37 (trehalase) were enriched in LW (adjusted P < 0.05 and |log2FC|> 1) (Fig. 3C).

### Metagenome-assembled genomes analysis

#### Taxonomic annotation of MAGs

**Figure 4:**
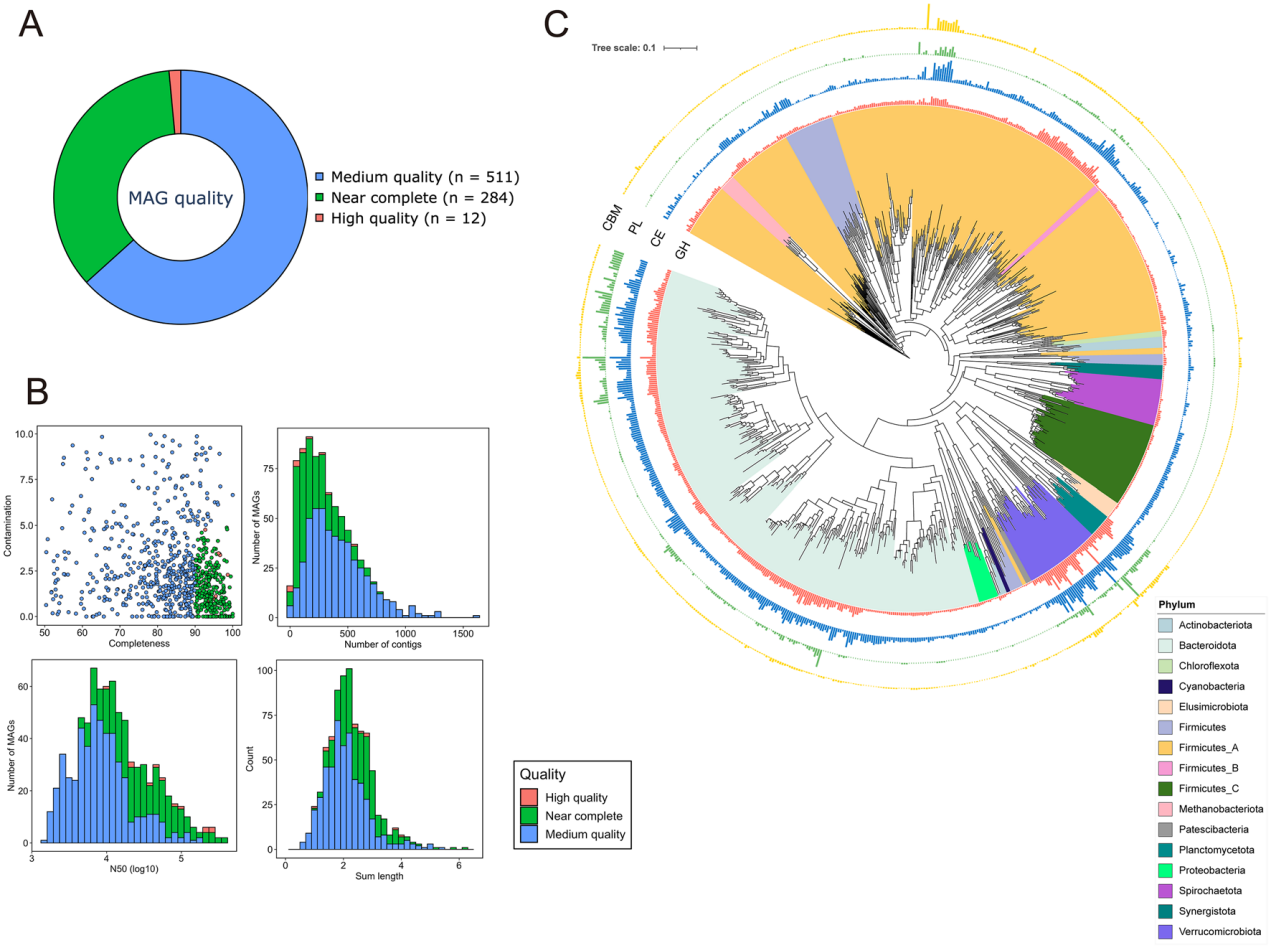
Overview of non-redundant MAGs in the rumen of Japanese Black cattle (ANI < 99%). (**A**) The pie chart shows the proportion of MAGs classified according to different criteria. High-quality MAGs had the 5S, 16S, and 23S rRNA genes, and at least 18 tRNAs with ≥ 90% completeness, ≤ 5% contamination. Near-complete MAGs indicate completeness ≥ 90% and contamination ≤ 5%. Medium-quality MAGs showed completeness ≥ 50% and contamination ≤ 10%. (**B**) The scatter plot indicates completeness and contamination statistics. Histograms show the distributions of N50 values, genome sizes, and number of contigs. (**C**) Phylogenetic tree of MAGs created using PhyloPhlAn v3.0.60^52^. MAGs with insufficient marker genes were excluded from analysis. Visualization was performed using iTOL^53^.

From our metagenome dataset (n = 37), 1,262 MAGs were reconstructed, with 1,003 MAGs met the threshold of ≥ 50% completeness and ≤ 10% contamination. Following dereplication at 99% ANI, we collected 807 non-redundant MAGs. These MAGs included 12 high-quality, 284 near-complete, and 511 medium-quality, with an average completeness of 84.5% and average contamination of 2.5% (Fig. 4A,B). The genome sizes of these MAGs ranged from 0.592 to 6.29 Mb, with N50 values between 1570 and 407,204 bp (Fig. 4B). When comparing 807 MAGs with RMGs at 95% ANI, 226 MAG remained representative. This suggested that the MAGs significantly expanded the collection of ruminal microbial genomes to the species level (Supplementary data S3). Only a few hundred MAGs are reconstructed from cattle rumen in Japan^12,50^. Although increasing the number of rumen MAGs was not the primary objective of this study, these MAGs will prove invaluable for metagenomic and metatranscriptomic analyses of the rumen microbiome, particularly in Japanese Black cattle.

According to the GTDB-Tk assignments, all 807 MAGs were assigned to the family level. However, two (MAG 727 and MAG 526) and 206 MAGs were not classified at the genus and species levels, respectively. This suggested that the MAGs represent novel genera and species (Supplementary data S3). Most MAGs belonged to Firmicutes_A (294 MAGs) and Bacteroidota (286 MAGs) (Fig. 4C). Other bacterial MAGs were classified as Firmicutes_C (44 MAGs), Firmicutes (40 MAGs), Verrucomicrobiota (39 MAGs), Spirochaetota (24 MAGs), Proteobacteria (15 MAGs), Planctomycetota (12 MAGs), Actinobacteriota (11 MAGs), Patescibacteria (10 MAGs), Elusimicrobiota (nine MAG), Synergistota (seven MAGs), Firmicutes_B (three MAGs), Chloroflexota (three MAGs), and Cyanobacteria (two MAGs). At the genus level, *Cryptobacteroides* (65 MAGs) and *Prevotella* (51 MAGs) in Bacteroidota were the predominant genera, which is consistent with a previous study^50^ (Supplementary data S3). All archaeal MAGs were taxonomically assigned to the phylum Methanobacteria (eight MAGs). Among these, six and two MAGs were classified as *Methanobrevibacter_A* and *Methanobrevibacter*, respectively. *Methanobrevibacter* spp. are dominant methanogens in the rumen^66^.

#### Functional analysis of MAGs

The genomic potential for VFA production is summarized in Supplementary data S4. A total of 694 MAGs (86.0%) had the potential to produce acetate, with the exception of Firmicutes_C and Cyanobacteria, which lacked genes related to acetate production. In addition, 333 (41.3%) and 360 MAGs (44.6%), primarily belonging to Bacteroidota and Firmicutes_A, respectively, contained genes associated with propionate and butyrate production. Notably, all MAGs from Firmicutes_C possessed genes encoding enzymes related to propionate production, which is consistent with the findings reported by Sato et al*.*^50^. All archaeal MAGs carried genes related to the CO_2_/H_2_ methanogenesis pathway (Supplementary Fig. S7). Moreover, MAGs 283 and 374 possess *mtaA*, *mtaB*, and *mtaC* genes, supporting methanogenesis from methanol. This indicated the potential of methanol to produce methane (Supplementary Fig. S7). These results indicated that all archaeal MAGs in this study were methanogens.

In total, 65,111 CAZyme domains were identified in the MAGs, with an average of 80.6 ± 2.06 domains per MAG (mean ± SE). The number of domains varied from 0 to 454 (Fig. 4C; Supplementary data S3). Several MAGs belonging to Bacteroidota, Firmicutes_A, Verrucomicrobiota, and Planctomycetota exhibited high abundances of GHs. The top 10 MAGs with the most abundant GHs were eight Verrucomicrobiota, one Planctomycetota (MAG 494), and one Bacteroidetes (MAG 531). Among the GH and carbohydrate esterase (CE) domains, oligosaccharide-degrading families, such as GH2, GH3, and GH31, debranching enzymes, including GH77 and GH23, and xylan esterases (CE1 and CE4) were widely distributed in our MAGs (Supplementary data S5). This suggested that CAZymes play essential roles in lignocellulose degradation in the rumen. A total of 1701 PULs were identified within 249 MAGs, the majority of which belonged to the phylum Bacteroidota, including 64 *Cryptobacteroides*, 51 *Prevotella,* and 46 *Sodaliphilus* (Supplementary data S3). The highest number of PULs identified within a single MAG was 37, observed in *Prevotella* (MAG 556), followed by 26 and 24 PULs within *Cryptobacteroides* MAGs (MAG 333 and MAG 572, respectively). Of the 1701 PULs, 755 contained various GHs and CEs, indicating the involvement of MAGs harboring these PULs in carbohydrate degradation in the rumen (Supplementary data S6).

#### Relationship between carcass traits and MAGs

**Figure 5:**
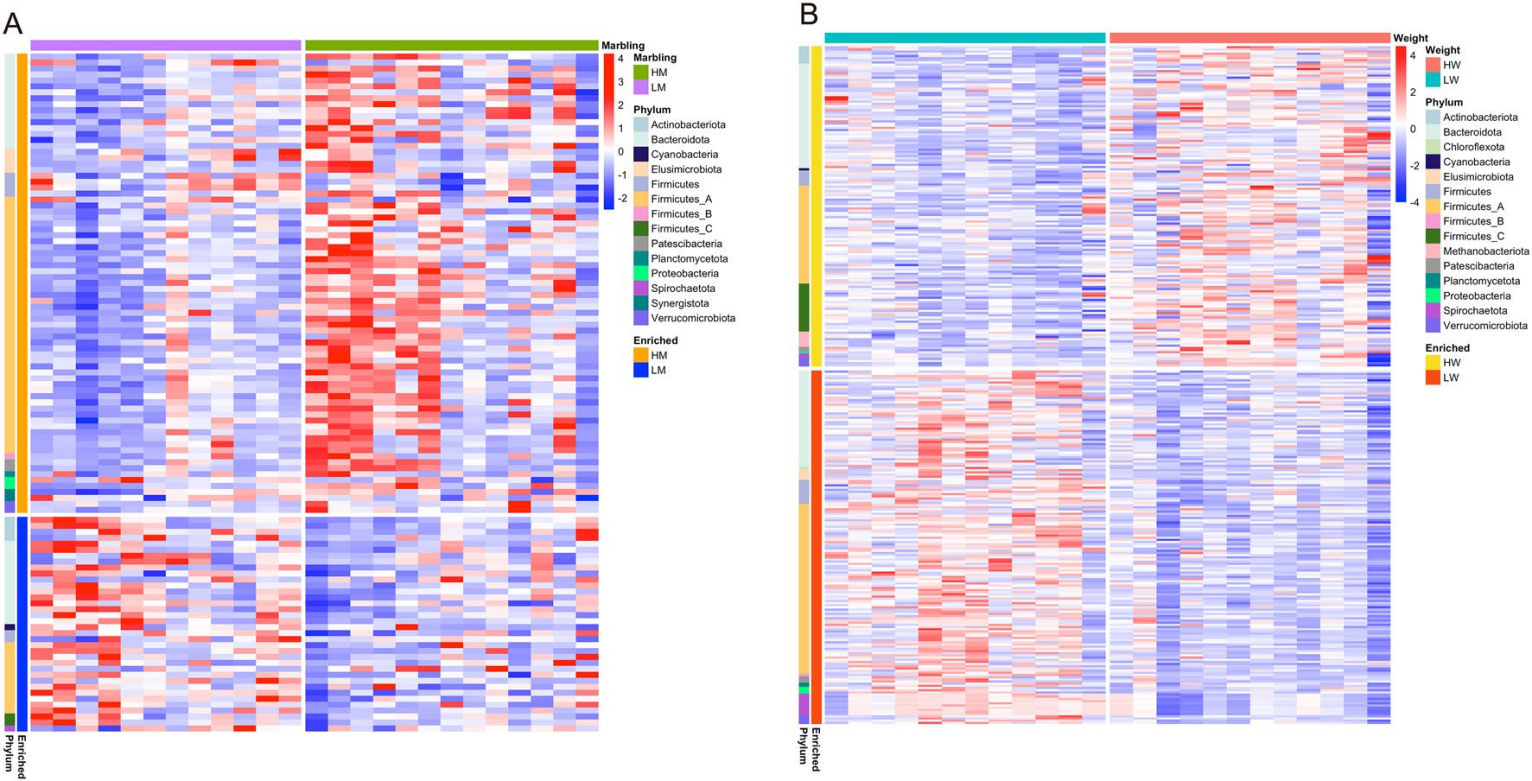
Heatmap showing the differences in MAGs. The heatmap shows MAGs with differential abundances between the (**A**) HM and LM groups and (**B**) HW and LW groups. Abundance was calculated using logarithmically transformed TPM values (log_10_(TPM + 1)). The color code represents the row Z-score. Significant differences were identified using DESeq2 (adjusted P < 0.05 and |log2 fold change|> 1).

In comparison between HM and LM cattle, 77 MAGs were enriched in HM and 36 were enriched in LM (adjusted P < 0.05, |log2FC|> 1) (Fig. 5A). Among these, 43 Firmicutes_A MAGs (55.8%) exhibited higher abundance in HM cattle, whereas only 12 Firmicutes_A MAGs (33.3%) were enriched in LM cattle. Firmicutes are associated with fat deposition in mice^72^. Similarly, in ruminants, the relative abundance of Firmicutes was higher in high-marbling sheep than in low- or middle-marbling sheep^9^. Thus, Firmicutes_A MAGs in the rumen may play a pivotal role in increasing the IMF content in beef cattle. Additionally, Elusimicrobiota (four MAGs), Patescibacteria (two MAGs), Planctomycetota (one MAG), Proteobacteria (two MAGs), Synergistota (two MAGs), Verrucomicrobiota (two MAGs) were exclusively enriched in HM cattle, while Actinobacteriota (four MAGs), Cyanobacteria (one MAG), Firmicutes_C (two MAGs), and Spirochaetota (one MAG) were specifically enriched in LM cattle (Supplementary data S3). Notably, no *Prevotella* MAGs displayed higher abundance in HM cattle, whereas seven MAGs were enriched in LM cattle (Supplementary data S3). Moreover, *Prevotella* MAG (MAG 556) and *Cryptobacteroides* MAG (MAG 572), which possessed 37 and 24 PULs, respectively, were more abundant in the LM cattle (adjusted P < 0.05, |log2FC|> 1). These findings suggested that the rumen microbiota in LM may exhibit greater carbohydrate metabolism.

When comparing HW with LW cattle, 147 MAGs were enriched in HW and 162 were enriched LW (adjusted P < 0.05, |log2FC|> 1) (Fig. 5B). Notably, seven *Methanobrevibacter_A* and *Methanobrevibacter* classified MAGs demonstrated higher abundance in HM than in LM cattle. This result is consistent with that of the protein-based analysis described earlier. Additionally, Firmicutes_C MAGs (22 MAGs), including *Selenomonas* (*Selenomonas_A*, *Selenomonas_B*, and *Selenomonas_C*, according to the GTDB) and *Succiniclasticum,* associated with propionate production*,* were exclusively enriched in HW cattle. *Succiniclasticum*^73^ and *Selenomonas*^74^ can utilize succinate to produce propionate via the succinate pathway in the rumen. Spirochaetota, specifically assigned to *Treponemataceae* and *Sphaerochaetaceae*, possesses various GHs and plays a considerable role in carbohydrate degradation and sugar metabolism in the rumen^75^. Notably, among Spirochaetota MAGs, *Treponema_D* MAGs (MAG 342 and MAG 593), possessing various GHs (Supplementary data S5), were enriched in the HW cattle, whereas CADAEX01 MAGs (10 MAGs), with fewer GHs, were enriched in the LW cattle (adjusted P < 0.05, |log2FC|> 1). Additionally, *Prevotella* MAG (MAG 556) and *Cryptobacteroides* MAG (MAG 572) were more abundant in the HW group (adjusted P < 0.05 and |log2FC|> 1). These results, in conjunction with the protein-based analysis, suggested that a higher polysaccharide-degrading capacity may be associated with improved carcass weight.

Recently, several researchers suggested that the rumen microbiome could be heritable^76,77^, and microbiota-driven breeding can improve beef quality^78^. Therefore, MAGs enriched in HM or HW cattle could potentially serve as biomarker candidates for genetic selection to increase marbling and carcass weight, respectively. However, 32 HW-enriched MAGs were more abundant in the LM cattle, whereas 62 LW-enriched MAGs were more abundant in the HM cattle. This suggested the possibility of decreased marbling or carcass weight when using MAGs as biomarkers for genetic selection. Therefore, careful consideration is required when applying microbiological genetic selection to the rumen.

One of the limitations of the study is missing data on several factors such as dietary feeds, age, and sex of the animals. Therefore, we cannot exclude the possibility that factors other than carcass traits affect the rumen microbiome. Future large-scale studies are required to validate the findings of this study.

## Conclusions

In summary, the current study revealed that the composition and function of the rumen microbiome of beef cattle are associated with carcass traits, such as marbling and carcass weight, via amplicon and shotgun sequencing. Notably, the abundance of methanogens, particularly *Methanobrevibacter* spp., differed between high- and low-carcass-weight cattle, resulting in differences in methane metabolism within the rumen. Furthermore, this study highlighted the involvement of several MAGs, most of which play essential roles in carbohydrate metabolism in the rumen, in relation to marbling and carcass weight in beef cattle. MAGs may play a pivotal role in improving both the quantity and quality of meat.

### Supplementary Information

Supplementary Information 1.

Supplementary Information 2.

Supplementary Information 3.

Supplementary Information 4.

Supplementary Information 5.

Supplementary Information 6.

Supplementary Figures.

## Data Availability

Raw sequence data were deposited in DDBJ (accession number DRA017253). non-redundant MAGs are available in figshare (https://figshare.com/s/8f4e195837347d36e7b0).
