# Supplementary material for: Impact of rumen microbiome on cattle carcass traits
Source: Sci Rep. 2024 Mar 13;14:6064. doi: 10.1038/s41598-024-56603-3 (PMC10937913; doi:10.1038/s41598-024-56603-3)
Supplement: Supplementary file 7 — Supplementary Figures. [file 41598_2024_56603_MOESM7_ESM.pdf]

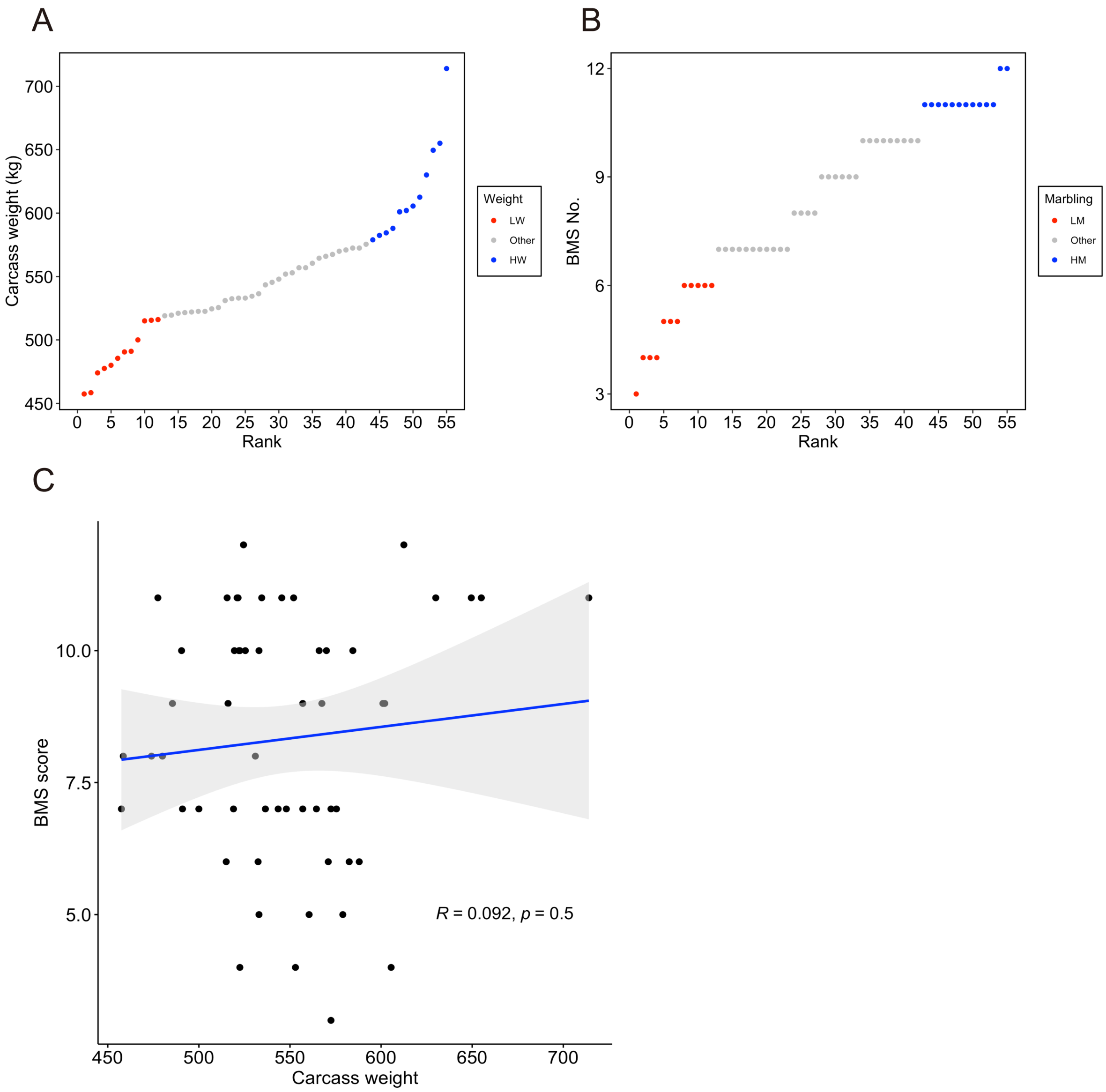

Supplementary Fig. S1 Carcass weight and BMS scores of the 55 Japanese Black cattle.

(A) Carcass weight and (B) BMS number in the animals. "Other" represents samples other than HW, LW, HM, and LM. (C) Correlation between carcass weight and BMS number.

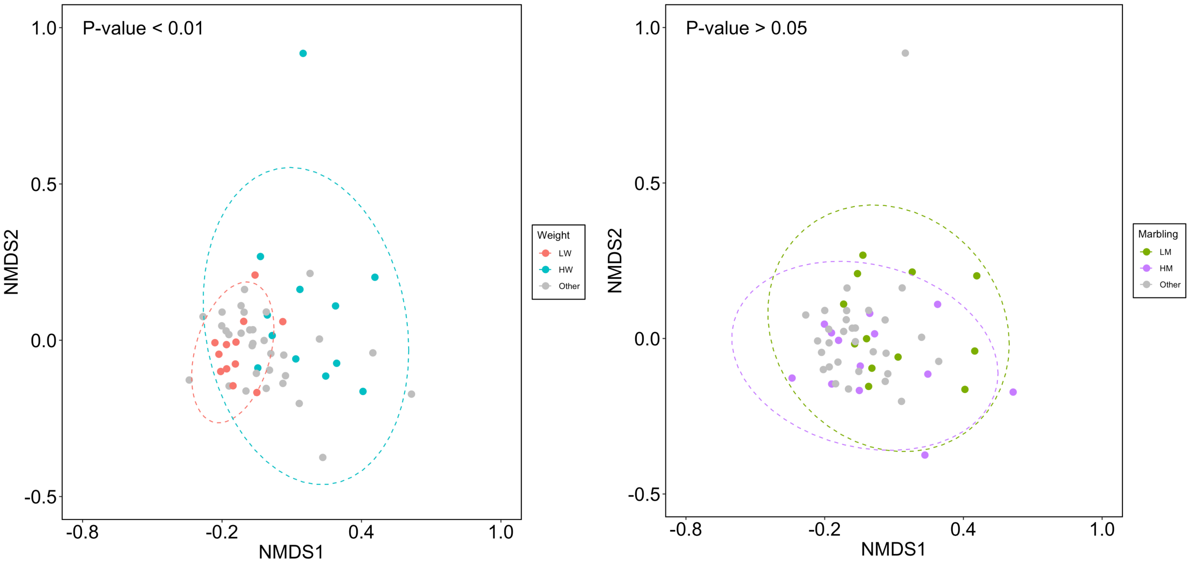

Supplementary Fig. S2 NMDS plots depicting Bray-Curtis dissimilarities at the genus level based on 16S rRNA amplicon sequencing. Significance was analyzed using PERMANOVA with 9,999 permutations. Ellipses represent 95% confidence intervals. "Other" represents samples other than HW, LW, HM, and LM.

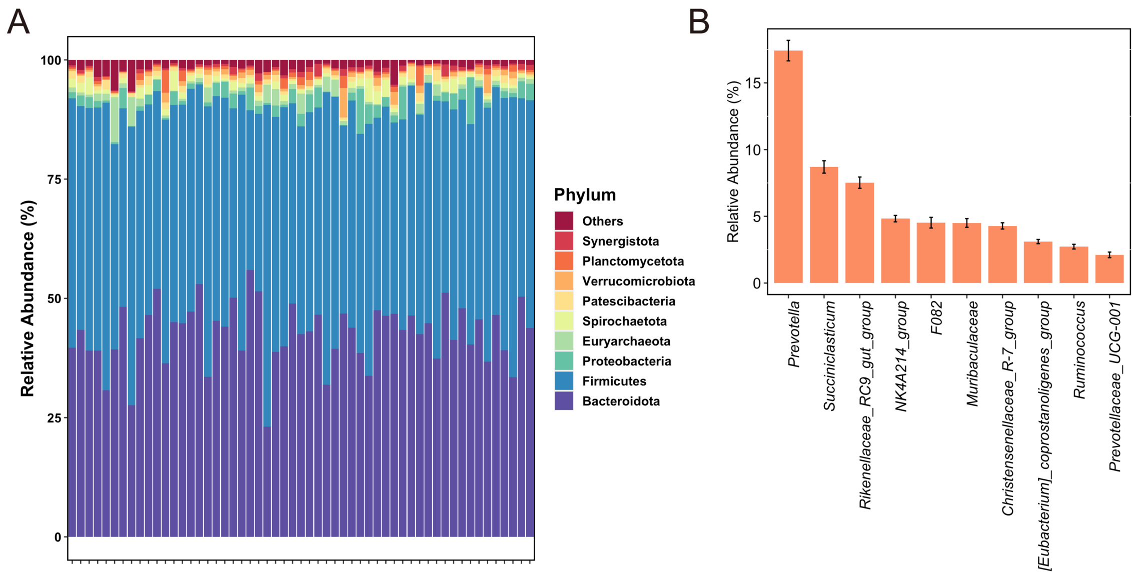

Supplementary Fig. S3 Taxonomic composition of the rumen microbiome in cattle based on 16S rRNA amplicon sequencing. (A) Relative abundance of taxa at the phylum level, and (B) the top 10 predominant genera.

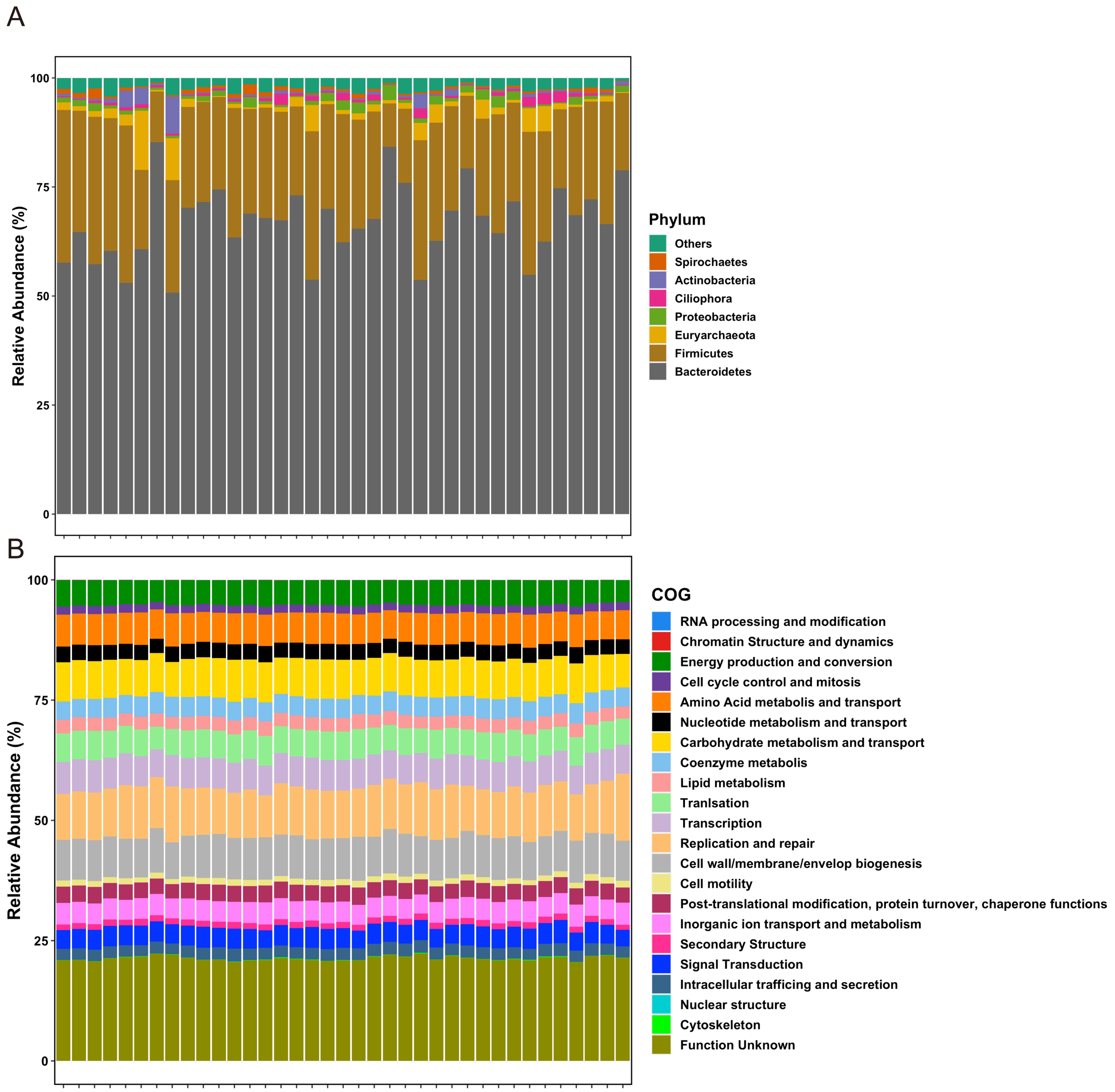

Supplementary Fig. S4 Taxonomic and functional profiles of the rumen microbiome in cattle by protein-based analysis using metagenomic sequencing. (A) Relative abundance of taxa at the phylum level, and (B) COG functional categories.

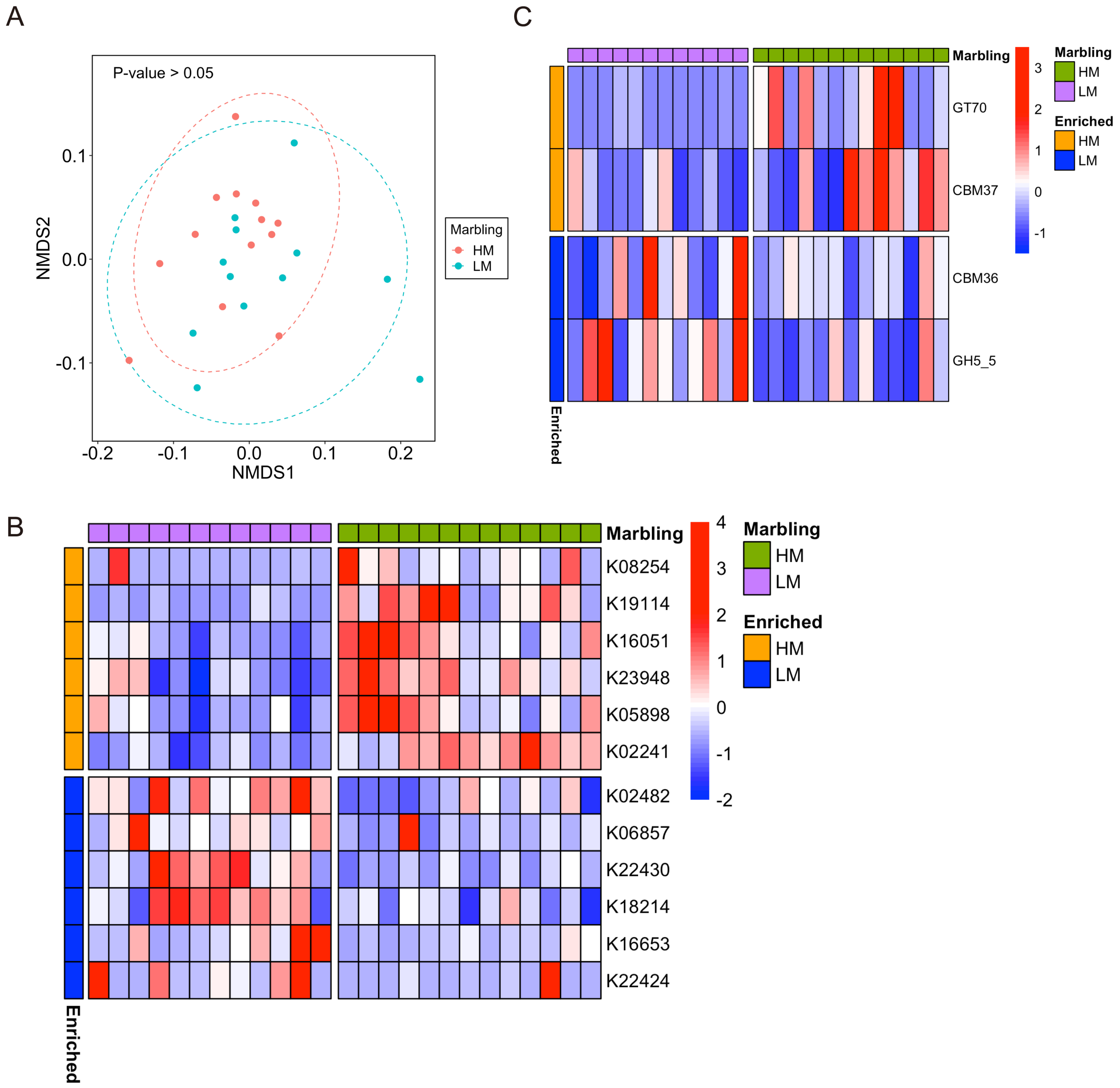

Supplementary Fig. S5 Functional differences in the rumen microbiome between HM and LM cattle revealed by protein-based analysis using metagenomic sequencing. (A) NMDS plots depicting Bray-Curtis dissimilarities of KO. Significance was analyzed using PERMANOVA with 9,999 permutations. Ellipses represent 95% confidence intervals. The heatmap shows the differential abundance of (B) KO and (C) CAZyme domains based on logarithmically transformed TPM values (log10 (TPM +1)). The color code represents the row Z-score. Significant differences were identified using DESeq2 (adjusted $P < 0.05$ and $|\log2 \text{ fold change}| > 1$).

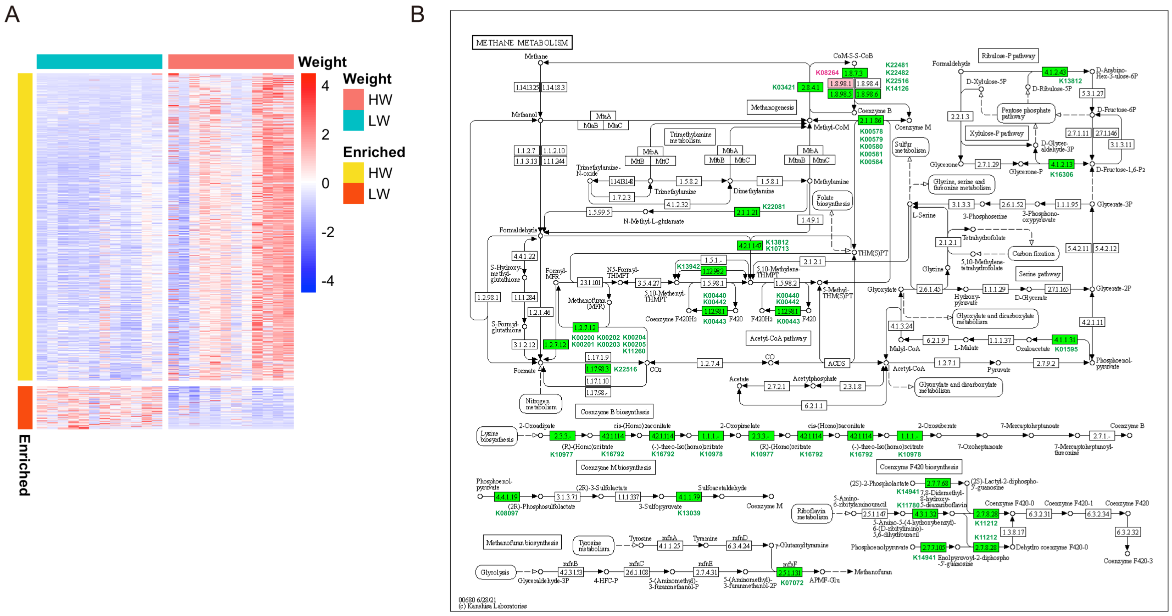

Supplementary Fig. S6 Functional differences in the rumen microbiome between HW and LW revealed by protein-based analysis using metagenome sequencing. (A) The heatmap depicts the abundance of KOs that showed significant differences in HW and LW based on logarithmically transformed TPM values (log10(TPM +1)). The color code represents the row Z-score. (B) Methane metabolic pathway (ko00680) visualized using KEGG mapper [34]. Green and pink indicate enrichment in HW and LW, respectively. Significant differences were identified using DESeq2 (adjusted P < 0 .05 and |log2 fold change| > 1).

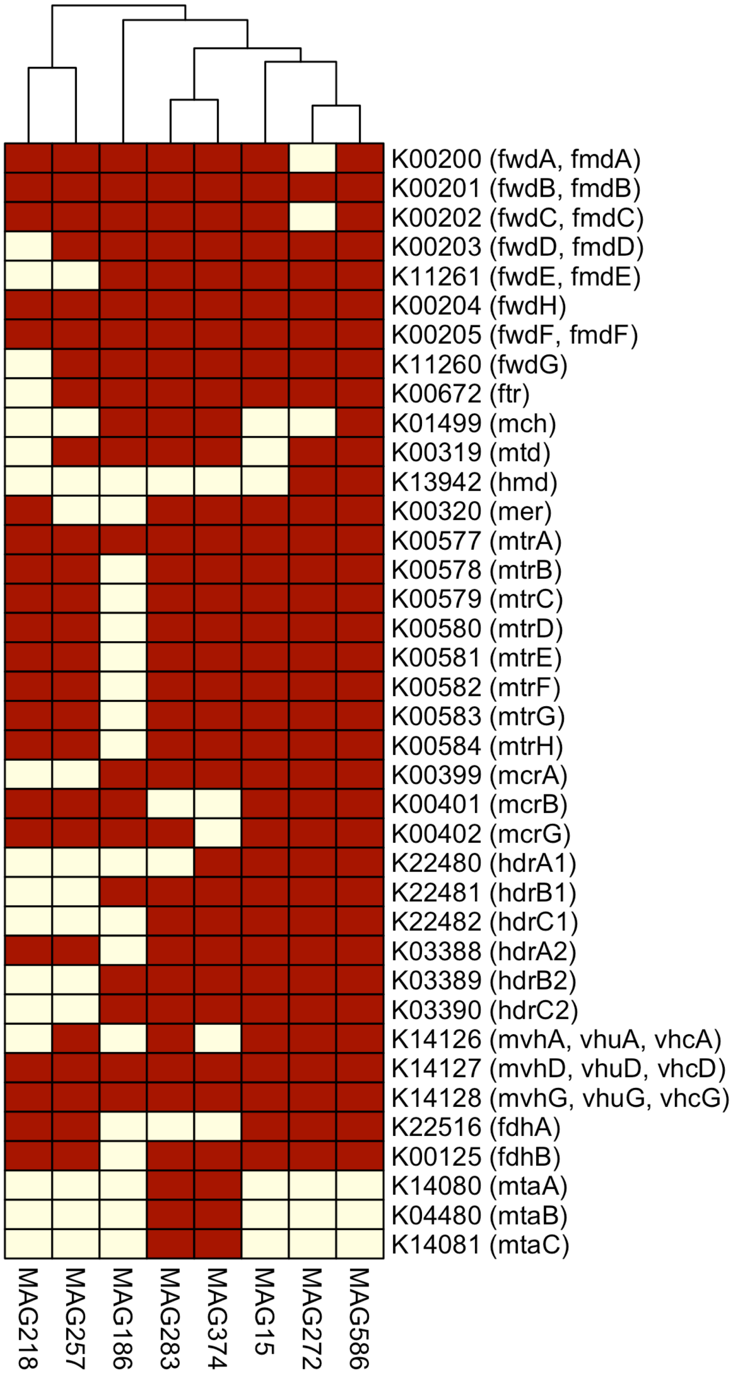

Supplementary Fig. S7 Potential to produce methane of archaeal MAGs. The row indicates KEGG orthology related to methanogenesis and the column represents MAGs. Presence and absence are depicted in dark red and light yellow, respectively.
